# Are health websites credible enough for elderly self-education in the most prevalent elderly diseases?

**DOI:** 10.1186/s12911-021-01397-x

**Published:** 2021-01-28

**Authors:** Saeideh Valizadeh-Haghi, Shahabedin Rahmatizadeh, Ali Soleimaninejad, Seyedeh Fatemeh Mousavi Shirazi, Parisa Mollaei

**Affiliations:** 1 Department of Medical Library and Information Sciences, School of Allied Medical Sciences, Shahid Beheshti University of Medical Sciences, Tehran, Iran; 2 Department of Health Information Technology and Management, School of Allied Medical Sciences, Shahid Beheshti University of Medical Sciences, Tehran, Iran; 3 Master of Geriatric Health, School of Public Health and Safety, Shahid Beheshti University of Medical Sciences, Tehran, Iran

**Keywords:** Geriatric diseases, Health portals, Patient education, Health websites, Website evaluation, Health information, E-health

## Abstract

**Background:**

The Elderly and their caregivers need credible health information to manage elderly chronic diseases and help them to be involved in health decision making. In this regard, health websites are considered as a potential source of information for elderlies as well as their caregivers. Nevertheless, the credibility of these websites has not been identified yet. Thus, this study aimed to evaluate the credibility of the health websites on the most prevalent chronic diseases of the elderly.

**Methods:**

The terms “Chronic obstructive pulmonary disease”, “Alzheimer's”, “Ischemic heart disease”, and “Stroke” were searched using the three popular search engines. A total of 216 unique websites were eligible for evaluation. The study was carried out using the HONcode of conduct. The chi-square test was carried out to determine the difference between conforming and nonconforming websites with HONcode principles and website categories.

**Results:**

The findings showed that half of the evaluated websites had fully considered the HONcode principles. Furthermore, there was a significant difference between websites category and compliance with HONcode principles (*p* value < .05).

**Conclusion:**

Regarding the poor credibility of most prevalent elderly diseases’ websites, the potential online health information users should be aware of the low credibility of such websites, which may seriously threaten their health. Furthermore, educating the elderly and their caregivers to evaluate the credibility of websites by the use of popular tools such as HONcode of conducts before utilizing their information seems to be necessary.

## Background

The rapid growth of the elderly population is a major issue in many countries. The increase in life expectancy has led to an increase in the number of elderly people [1]. According to WHO definition, people over 65 are considered as elderly population [2]. Epidemiological evidence suggests that the risk of developing chronic diseases increases with aging [3]. According to the evidence provided by the Institute for Health Metrics, the chronic diseases including Ischemic heart disease (IHD), stroke, Alzheimer's disease, and Chronic obstructive pulmonary disease (COPD) are the top four most prevalent chronic diseases of the elderly which are causing death worldwide [4, 5]. Therefore, the dramatic growth of the elderly population is a major challenge for seniors and their families, as well as their community in which they live [6].

Population aging imposes significant economic burdens on health systems [6, 7], as chronic diseases usually force the elderly to use health services and care, including home care and hospitalization [8]. Approximately 23% of the disease burden is attributed to people aged 65 years or above [9]. Therefore, actions should be taken to reduce the burden of geriatric diseases by encouraging the participation of the elderly and their caregivers in health-related decision-making and disease management [10]. However, such actions require adequate health information and literacy. People who have a high level of knowledge and understanding of the relevant health information, are more informed and are more health literate. A higher level of health literacy has major advantages, such as improvement of self-care skills in the elderly [11, 12], information acquisition about chronic diseases [13], and appropriate and timely use of preventive health services [14]. Inadequate health literacy is associated with poor self-management abilities in a wide range of older adults [15], poor physical health [15], the highest mortality rate [14], and frequent readmission among elderlies [16]. As a result of these benefits and also disadvantages, people commonly obtain complementary health information from sources other than health professionals [17].

In this regard, the Internet is a potential source of information for obtaining health information [18]. The medical information available on the Internet is increasingly used by the elderly and their caregivers [19–21]. Fox (2012) believes that elderly caregivers use the Internet to deliver home health care [19].

Evidence shows that the information available on the Internet influences the individuals’ health behaviors, treatment choices, and healthcare decision makings [22] and encourages them to involve in the process of self-care through improving the individuals` understanding of their own condition [23]. Moreover, it can empower patients [24] especially older adults [25] to play a more active role in disease management. Increased patient empowerment and playing an active role in disease management can help the elderly cope better with their disease which can lead to minimizing the burden of disease on healthcare system and society.

Nevertheless, the credibility of health websites is a concerning issue and it is highly possible to find incomplete and inaccurate health information on the Internet [26–28]. In a study by Butler, patients with low back pain were advised not to use the Internet as a source of information unless the website was found to be evidence-based [29]. Several institutions have developed tools to help evaluate the credibility of websites providing health information, including JAMA [30], DISCERN [31], and HONcode [32]. However, the credibility of the websites related to the most prevalent chronic diseases of the elderly has not been studied. Accordingly, access of the elderly and their caregivers to reliable websites is important. These websites help them to obtain reliable information and to be active in disease management as well as prevent them from severe risks to the treatment and recovery processes [33]. However, little is known about the credibility of websites for the elderly and their caregivers concerning the most prevalent elderly disease. Thus, this study aimed to evaluate the credibility of health websites containing health information related to the top four most prevalent deadly diseases in the elderly [4, 5] which are available through general search engines. Furthermore, this study aimed to find out the relationship between credibility and search result page as well as categories of retrieved websites.

## Methods

Search engines play an important role in obtaining health information by non-specialists [34]. Thus, to simulate the real searching environment, three most frequently used search engines i.e. Google, Yahoo, and Bing [35, 36] were selected to conduct the study.

We investigated the credibility of websites related to the top four most prevalent elderly diseases as they make 52% of all death in the elderly [5]. Regarding that patients are confused with medical terms [37], the CDC Plain Language Thesaurus [37] was checked for the plain terms that lay people likely use. The search terms corresponding to each disease (COPD; Alzheimer's; IHD; Stroke) were: “Chronic obstructive pulmonary disease”, “Alzheimer's”, “Ischemic heart disease”, and “Stroke”. The search was performed using Google Chrome on December 28, 2019.

The browsing history, cookies, and cached images were removed prior to each search process. Default search engine settings were used, producing 10 website results per search. Since 90% of search engine users only explore the first three pages of the search results [38], the first 30 websites returned by each search engines for each keyword were selected for study (90 websites for each keyword, a total of 360 websites). Websites were eligible for inclusion if they were: (1) in English, (2) free to access, and (3) provided information on the most prevalent elderly diseases associated with the search terms.

Websites were excluded if they were academic articles, academic journal websites, non-English-language, duplicate websites, password-protected, inaccessible links (dead links), advertisement sponsored links, and contained information irrelevant to search terms.

**Fig. 1:**
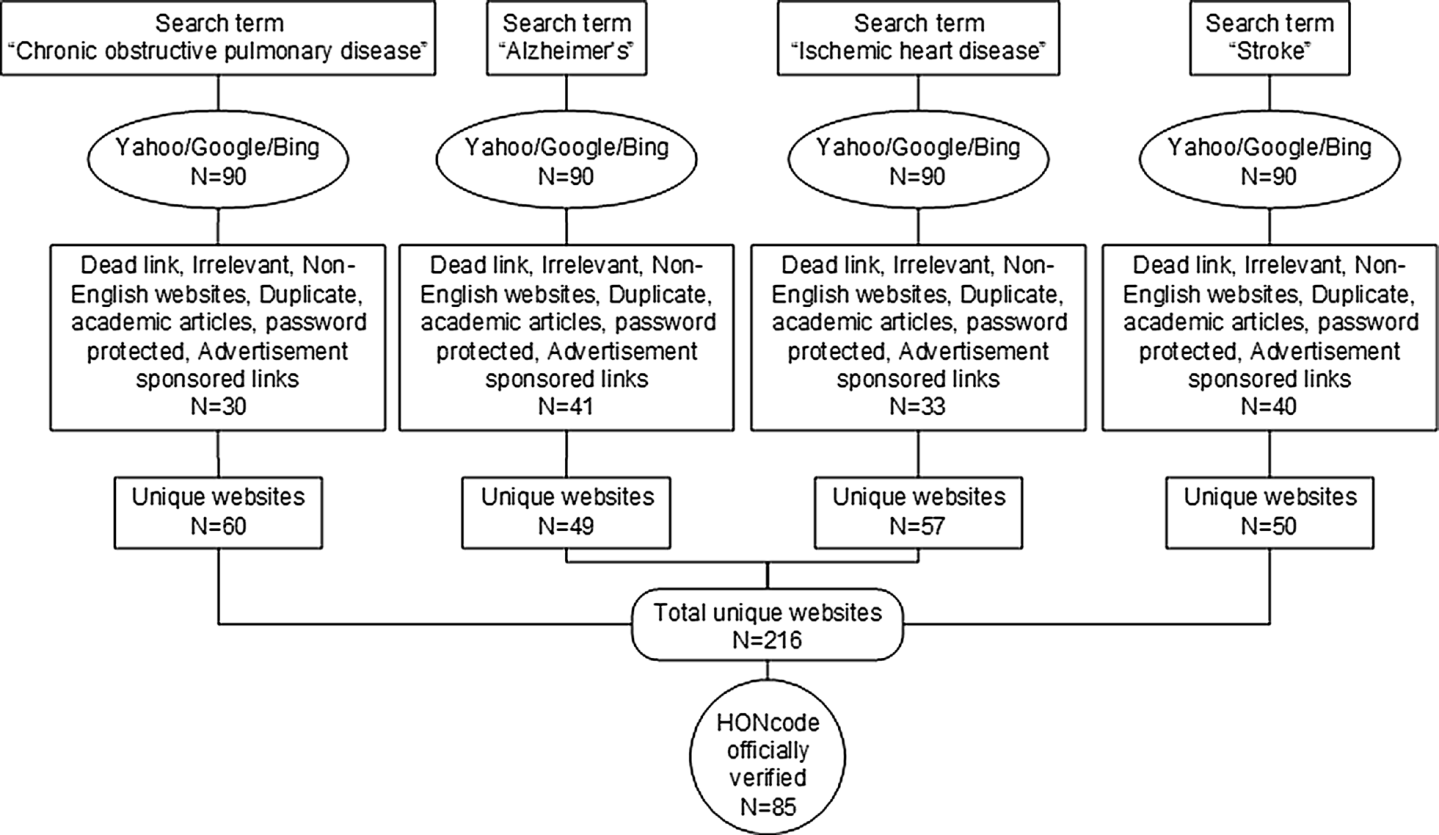
The internet search flow diagram

After excluding 144 websites, the remaining 216 unique websites were evaluated by direct observation. The number of unique websites for each disease and the search process is demonstrated in Fig. 1. The selected websites were divided into three categories: organizational, commercial, and governmental.

### Website frequency and affiliation

Based on the website domain and the aim of the website, the selected websites were divided into three categories including Governmental, Commercial, and Organizational Websites. Governmental websites included websites that are run by government organizations such as the NHS (National Health Service). Commercial websites refer to websites that buy, sell, or provide a service for a fee. Organizational websites included ones that belong to charities or communal organizations.

### Data collection tools

In this study, the HONcode of conduct was employed to assess the credibility of the health websites on the most prevalent diseases of the elderly. This tool consists of eight criteria: Authority, Complementarity, Privacy, Attribution, Justifiability, Transparency, Financial Disclosure, and Advertising Policy (Table 1). Health organizations can apply for the HONcode certification to verify the credibility of their websites. The certified websites can display the HONcode logo on their webpage. This tool was selected to carry out the present study because it is reliable and has been widely used in several studies to evaluate the credibility of health websites on various topics [27, 40–45].

**Table 1:** HONcode principles

Criterion Name	Criterion Definition
Authority	Indicate the qualifications of the authors
Complementarity	Information should support, not replace, the doctor-patient relationship
Privacy	Respect the privacy and confidentiality of personal data submitted to the site by the visitor
Attribution	Cite the source(s) of published information, date medical and health pages
Justifiability	The site must back up claims relating to benefits and performance
Financial disclosure	Identify funding sources
Advertising policy	Clearly distinguish advertising from editorial content

The table information is adapted from the HONcode website [39]

An 8-question checklist was developed based on HONcode criteria. Each of the questions can receive a score from 0 to 1, corresponding to a non-conforming and conforming website with each criterion, respectively. A website would be considered credible if comply with all the eight criteria. Moreover, the official HONcode toolbar was used to identify websites that were officially certified and approved by the Health on the Net Foundation. The toolbar is available through the official website of Health on the Net Foundation [46].

### Process

All of the selected websites were evaluated manually by three screeners who were specialists in geriatric health (A.S., F.M., and P.M.) and reached consensus on their HONcode scores for each website. These 3 raters and the evaluation process were supervised by S.V. (Health information and Medical library specialist) and S.R. (Medical Informatics specialist) to ensure the accuracy of the obtained data. Cohen's κ was run to determine if there was agreement between two supervisors on whether 30 websites were exhibiting credible or not. There was high agreement between the two supervisors’ judgements, κ = 0.733, *p* < .0005.

The Chi-square test was carried out to determine any difference between conforming and nonconforming websites with HONcode principles and search result page as well as the categories of retrieved websites. Furthermore, the Chi-square test was carried out to find any difference between the proportion of websites in compliance with HONcode principles by diseases.

An alpha level of 0.05 was applied for all statistical tests. The collected data were analyzed using SPSS version 18.

## Results

**Fig. 2:**
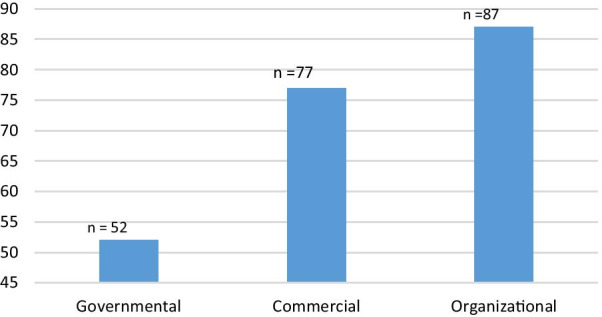
The distribution of websites on the most prevalent elderly diseases

The frequency of retrieved websites is shown in Fig. 2. Most of the retrieved websites were organizational (40.3%), and the minority (24.1%) were governmental. The findings revealed that 108 out of 216 websites (50%) are completely in compliance with HONcode principles. Amongst these, 78.7% were officially approved by HON Foundation and presented the HONcode seal of approval.

The compliance of the surveyed websites with the eight HONcode criteria is shown in Table 2. The highest compliance with the criteria was related to Transparency (98.1%), which was fully met in websites retrieved by Bing search engines (100%), while the lowest compliance was attributed to the Authority (64.8%).

**Table 2:** The total number and percentage of websites that comply with each criterion of HONcode

Criteria	Search engines			Total N = 216
	Google N = 85	Yahoo N = 65	Bing N = 66	
Authority	55 (58.8%)	42 (64.6%)	43 (65.2%)	140 (64.8%)
Complementarity	67 (78.8%)	52 (80.0%)	49 (74.2%)	168 (77.8%)
Privacy	79 (92.9%)	64 (98.5%)	61 (92.4%)	204 (94.4%)
Attribution	73 (85.9%)	59 (90.8%)	58 (87.9%)	190 (88.0%)
Justifiability	76 (89.4%)	62 (95.4%)	63 (95.5%)	201 (93.1%)
Transparency	84 (98.9%)	62 (95.4%)	66 (100%)	212 (98.1%)
Financial disclosure	70 (82.4%)	52 (80.0%)	57 (86.4%)	179 (82.9%)
Advertising policy	61 (71.8%)	42 (64.6%)	45 (68.2%)	148 (68.5%)

**Fig. 3:**
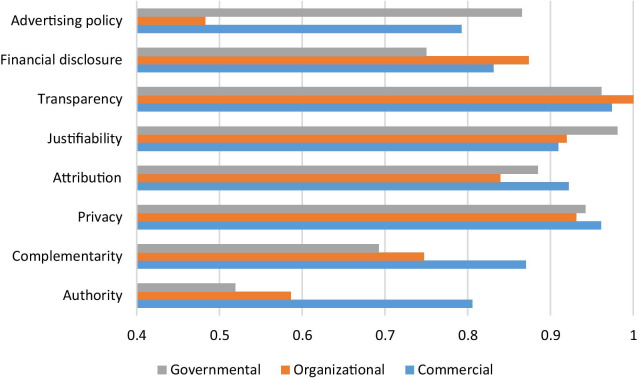
The adherence to the HONcode principles by each category of websites

The adherence of websites to each HONcode principle based on websites category is presented in Fig. 3. The findings revealed that the Advertising policy, Financial disclosure, and Transparency principles have been less considered in commercial websites rather than the governmental and organizational websites. Instead, the governmental websites have less considered the Authority, Complementarity, Transparency, and Financial disclosure rather than the other categories.

The chi-square test of independence revealed that there was no significant association between search results page and compliance with HONcode principles, *X*^2^ (2, *N* = 216) = 3.031, *p* value = .22.

Furthermore, the proportion of websites in compliance with HONcode principles did not differ by Search Engines, *X*^2^ (2, *N* = 216) = 0.088, *p* value = .957. A chi-square test of independence was performed to examine the relation between websites category and the websites in compliance with HONcode principles (Table 3). The findings revealed the significant relation between these variables, *X*^2^ (2, *N* = 216) = 11.03, *p* value = .004. Regarding the compliance with HONcode principles, commercial websites were significantly in better compliance comparing organizational (*p* value = .006) and governmental websites (*p* value = .003). Nevertheless, no significant relation was found between organizational and governmental websites (*p* value = .546). Furthermore, findings revealed that the proportion of websites in compliance with HONcode principles did not differ by diseases, *X*^*2*^ (*3, N* = 216) = 5.501, *p* value = .139.

**Table 3:** Chi-square test results for differences in conforming and nonconforming websites with HONcode principles in rating for search results attributes

Variables	Compliance with HONcode principles		*X*^2^	*df*	*p* value
	No	Yes			
Search results					
Page1	36 (42.9%)	48 (57.1%)	3.031	2	.220
Page2	39 (56.5%)	30 (43.5%)			
Page3	33 (52.4%)	30 (47.6%)			
Category					
Commercial	27 (35.1%)	50 (64.9%)	11.030	2	.004
Organizational	49 (56.3%)	38 (43.7%)			
Governmental	32 (61.5%)	20 (38.5%)			
Search engines					
Google	43 (50.6%)	42 (49.4%)	0.088	2	.957
Yahoo	33 (50.8%)	32 (49.2%)			
Bing	32 (48.5%)	34 (51.5%)			
Key words (disease)					
Chronic obstructive pulmonary disease	31 (51.7%)	29 (48.3%)	5.501	3	.139
Ischemic heart disease	22 (38.6%)	35 (61.4%)			
Alzheimer's	30 (61.2%)	19 (38.8%)			
Stroke	25 (50%)	25 (50%)			

## Discussion

To our knowledge, the present study is the first research to evaluate the credibility of health websites related to the most prevalent elderly diseases. The results showed that health websites (retrieved by general search engines) regarding the most prevalent geriatric diseases are not credible enough, which is in line with the results of other studies evaluating health websites on various topics [27, 33, 40–45, 47–52]. Out of 216 retrieved websites, only 39.4% were officially approved by Health on the Net Foundation. Moreover, the findings revealed that overall, 108 websites (50%) of the evaluated websites were in compliance with HONcode.

Access to credible health websites is essential for informing elderlies and their caregivers about the most prevalent elderly diseases, since it empowers them to cope with those diseases. Failure to comply with HONcode criteria indicates that seniors may encounter websites that are not reliable enough. These websites may contain inaccurate, misleading, and inadequate information, which can influence their preventive actions as well as their decision-making regarding the treatment choices of chronic diseases.

It’s worth noting that the findings revealed that there is a significant association between website category and compliance of websites with HONcode principles (*p* value = .004). While findings of a few pieces of research have indicated that commercial websites have lower credibility than other websites [53, 54]. The findings of the present study showed that most of the commercial websites are in compliance with HONcode principles and are credible than the websites in other categories (Table 3). Furthermore, the findings showed that governmental websites which generally contain credible educational information [55] were not completely in compliance with HONcode principles. This is important due to the importance of government websites as a source of health information for lay people. Thus, government websites must take substantial steps to improve the credibility of their websites regarding compliance with the HON principles. It is also worth noting that regarding that the origin of a website does not guarantee the higher quality of information available on that website [56], accordingly, the elderly and their caregivers are recommended to use online health information after consultation with a healthcare professional.

The compliance with the authority criterion represents the credibility of the information source since the information provided by experts is more reliable [57]. In the present study, only 64.8% of the surveyed websites had specified the name and expertise of the authors (Table 2). In similar studies evaluating websites on MERS disease [45], and websites on Ebola disease [44], the authority was the least considered criterion [58], while patients need sufficient information about the author’s identity to be able to assess the trustworthiness of information [48]. Therefore, health websites on the most prevalent elderly diseases need to pay more attention to this criterion to increase their trustworthiness for their readers.

The medical information on the Internet should not replace direct patient-physician relationship, since such information is intended to provide support and training for the readers and must not be a substitute for direct medical advice. The complementarity aspect of online medical information should be clearly stated on health websites. However, in the present study, only 77.8% of the evaluated websites complied with the complementarity principle, which most were commercial type websites (Table 2). Since only a small percentage of people tend to consult their physician about the health information obtained from the Internet [59], it is essential that health websites take this criterion into consideration so that the elderly and their caregivers utilize online health information with more caution after consulting a physician.

The justifiability criterion indicates that any information on a website, which defines the performance of a particular treatment, medication, or commercial medical device and refrains from discussing its side effects, is prohibited due to dishonesty in presenting information or commercial purposes. The findings of the present study showed that adherence to the justifiability principle is in good situation but still a small percentage of the websites were not in compliance with this principle (Table 2).

The distinction between commercial and scientifically edited content is another important criterion, which should be taken into consideration in health websites. If advertising is a source of funding for the website, the policy for presenting such content should be clearly stated. Moreover, the advertising content displayed on a website must be presented in a way that individuals can easily distinguish it from scientific and medical content. However, only 68.5% of the surveyed websites considered the advertising policy criterion. It should be noted that access to such websites may guide the elderly and their caregivers to unreliable and commercial information, which may threaten their health.

Moreover, websites should describe their privacy policy and define how they handle the users’ private information, such as email addresses and email content. The privacy policy is among seven core issues of website usability design and is of particular importance in creating effective websites [60]. Most websites assessed in this study had specified their privacy policy which is in line with the researches that have been carried out on other health topics [43, 44].

Based on the present findings, the transparency principle was considered in the majority of websites (98.1%). Thus, the elderly and their caregivers will be able to communicate with the content editors and webmasters in case of need for additional information.

According to the attribution principle, the publication date, as well as the most recent content updates, should be posted on the website. Adherence to this principle can help increase the credibility of health websites. The present study revealed that the attribution principle was considered in the majority of the websites related to common geriatric diseases (94.4%). Nevertheless, a small percentage of websites did not pay enough attention to this principle.

In the current study, the proportion of websites that were in compliance with HONcode principles did not differ by search engines as well as diseases (Table 3). While, in other studies on health websites related to periodontal disease and Ebola virus disease, the websites retrieved by Google were in greater compliance with the HONcode criteria [44, 61]. Furthermore, most of the websites that were appeared on the first page of search engine results were completely in compliance with HONcode principles, though, the difference was not statistically significant (Table 3).

In addition to the general public, all professional healthcare providers, including physicians, dentists, nurses, and public health workers, use the health information provided by search engines. A study by Hider et al. showed that 63% of specialists used Google at least once a month, while only 42% of them used medical information databases, such as Ovid and PubMed [62]. In this regard, healthcare providers should be aware of the poor credibility of health websites retrieved by public search engines so that they choose reliable medical databases.

Furthermore, healthcare providers must be aware that people use the Internet as a source of medical information. Therefore, they must advise and conduct their patients to use credible health websites and help them assess the quality of medical information available on the Internet [63] and encourage them to use online information only after consulting their physicians.

Finally, it is recommended that the content of websites related to the most common diseases of the elderly be evaluated, as the credibility of a website does not necessarily reflect the quality of information [64].

## Conclusion

The present study revealed that websites about the most prevalent geriatric diseases, retrieved by public search engines, are not credible enough, nevertheless, they may be used by seniors and their caregivers for obtaining health information. It is recommended that qualified geriatric health specialists guide the elderly and their caregivers to use trustable health websites for making proper disease about prevention and treatment choices. Moreover, it is necessary to educate the elderly regarding the evaluation tools of health websites to assess the credibility of websites before utilizing the provided content. In this regard, health libraries and medical librarians can play an important educational role. Health website owners, on the other hand, need to make strides to create reliable health websites by using approved tools such as HONcode criteria. Similarly, elderly care organizations are suggested to improve their website credibility and provide reliable information for seniors and their caregivers to prevent them from using unreliable websites. This may lead to help better disease management resulting in decreasing the burden of diseases in the community.

## Limitations

This study had some limitations. First, it was only conducted on websites regarding the most prevalent elderly diseases; therefore, the different results might be obtained for other elderly diseases. Additionally, search in other geographical areas and different time period can affect the present results. Moreover, if an individual applied a search engine other than the ones applied in this study, the results might be less applicable. Finally, websites are being constantly developed and updated; therefore, the present findings might have been affected.

## Data Availability

The datasets used and/or analyzed during the current study are available from the corresponding author on reasonable request.

## Abbreviations

IHD: Ischemic heart disease
COPD: Chronic obstructive pulmonary disease
HON: Health on the net foundation
